# The Apoptotic Effects of Toosendanin Are Partially Mediated by Activation of Deoxycytidine Kinase in HL-60 Cells

**DOI:** 10.1371/journal.pone.0052536

**Published:** 2012-12-27

**Authors:** Jianming Ju, Zhichao Qi, Xueting Cai, Peng Cao, Yan Huang, Shuzhen Wang, Nan Liu, Yijun Chen

**Affiliations:** 1 State Key Laboratory of Natural Medicines and Laboratory of Chemical Biology, China Pharmaceutical University, Nanjing, China; 2 Department of Pharmaceutical Analysis and Metabolomics, Jiangsu Province Academy of Traditional Chinese Medicine, Nanjing, China; Rutgers University, United States of America

## Abstract

Triterpenoid toosendanin (TSN) exhibits potent cytotoxic activity through inducing apoptosis in a variety of cancer cell lines. However, the target and mechanism of the apoptotic effects by TSN remain unknown. In this study, we captured a specific binding protein of TSN in HL-60 cells by serial affinity chromatography and further identified it as deoxycytidine kinase (dCK). Combination of direct activation of dCK and inhibition of TSN-induced apoptosis by a dCK inhibitor confirmed that dCK is a target for TSN partially responsible for the apoptosis in HL-60 cells. Moreover, the activation of dCK by TSN was a result of conformational change, rather than auto-phosphorylation. Our results further imply that, in addition to the dATP increase by dCK activation in tumor cells, dCK may also involve in the apoptotic regulation.

## Introduction

*Melia toosendan* Sieb. et Zucc. is a medicinal plant with analgesic, insecticidal and anti-inflammatory characteristics, which mainly grows in China and India [1]. Triterpenoid toosendanin (TSN, Figure S1), is a colorless and acicular crystal isolated from the barks or fruits of this medicinal plant. The pharmacological effects of TSN have been investigated since the elucidation of the chemical structure in 1975 [2] and the correction in 1980 [3]. TSN possesses a variety of biological activities. For example, TSN inhibits feeding and development of insects and is used as a botanical insecticide without residual toxicity and public harm in the fruit and vegetable production [4], [5]. More importantly, it is applied in the clinical practice as an anthelmintic vermifuge in China [6]. TSN can selectively block acetylcholine release from nerve terminals and exhibits antibotulismic effects *in vitro* and *in vivo* [7]–[9]. Recent studies indicate that TSN could be useful in the treatment of human cancers because it showed anti-proliferative effects on a number of human cancer cells and pro-apoptotic effects *in vitro* by Hoechst 33258 staining, DNA fragmentation analysis and flow cytometric analysis [10]–[12]. Furthermore, TSN also induces apoptosis in mouse hepatocellular carcinoma H22 cells [1] and causes cytochrome c release from mitochondria into cytosol, markedly activating caspase 3, 8, and 9, and leading to a decrease in the expression of anti-apoptotic protein Bcl-2 and an increase in the expression of pro-apoptotic protein Bax [1], [11]. Different from previous reports, when HL-60 cells were treated with TSN, suppression of JNK signaling pathway was identified to be responsible for the pro-apoptotic effects [13]. These findings suggested the complexity of the TSN-induced apoptosis, which may be mediated by targeting multiple signaling pathways.

Natural products have become important molecular probes to study different cellular processes by virtue of their ability to bind to specific protein targets and interfere with their cellular functions. Therefore, large efforts have been directed at the identification of biologically active targets of natural products. Notable examples include the identification of prohibitin 1 as the target of marine natural product aurilide [14], Xeroderma Pigmentosum B (XPB) as the target of immunosuppressive agent triptolide [15], splicing factor SF3b as a target of antitumor natural product pladienolide [16]. The discovery of these useful targets plays vital roles in the elucidation of molecular mechanism of biological processes.

In this study, we chose TSN as a molecular probe to capture its target protein in HL-60 cells by serial affinity chromatography. A specific binding protein was identified as deoxycytidine kinase (dCK). To establish the relationship between dCK and TSN-induced apoptosis in HL-60 cells, we have provided three lines of evidence: a) recombinant dCK was directly activated by TSN; b) LP-503392, a dCK inhibitor, significantly inhibited TSN-induced apoptosis; and c) mutagenesis studies indicated that dCK activation was most likely caused by conformational change instead of auto-phosphorylation. Collectively, our results suggested that TSN binds dCK to induce conformational change and subsequently activates dCK to partially contribute to the pro-apoptotic effects in HL-60 cells.

## Materials and Methods

### Synthesis of Toosendanin-succinate (TSN-S)

TSN-S was synthesized following the protocol reported previously (Figure S1) [17]. The modified compound with spacer arm of certain length was prepared to ensure the success of this research.

A mixture of TSN (0.5 mmol, Xi’an Insecticide Biological Projected Co., Ltd., Xi’an, China), succinic anhydride (0.5 mmol, Shanghai Chemical Reagent Co., LTD, Shanghai, China) and anhydrous sodium acetate (150 mg) in dry acetone (20 mL) was refluxed in 60°C water-bath for 10 h. The reaction mixture was filtered and the filtrate concentrated; the residue was then dissolved in 30 mL ethyl acetate. The solution was extracted with 3% Na_2_CO_3_ (3×30 mL), washed with 50 mL ethyl acetate and 50 mL ether, respectively. The aqueous phase was adjusted to pH 1–2 with 1 N HCl, extracted with 100 mL ether, dried over anhydrous Na_2_SO_4_ and concentrated under reduced pressure to obtain crude product, which was recrystallized to give 28-hemisuccinyl-TSN (TSN-S) as white crystals (110.0 mg, yield 38.3%). The structures was confirmed by comparison of its MS, NMR and IR spectral data with those in the literature [16]. Spectral data of TSN-S were the following: C_34_H_42_O_14_; SIMS m/z 673(M-1); ^1^H NMR (500 MHz, CDCl_3_) δ: 4.30 (1H, overlap),1.90 (1H, overlap) 2.78 (1H, m),5.25 (1H, m),2.68 (1H, m),1.73 (1H, m) 2.08 (1H, m),3.67 (1H, m),4.60 (1H, s),5.25 (1H, s),3.76 (1H, s),1.90 (1H, overlap) 2.23 (1H, dd, J = 13.3, 6.4),2.97 (1H, dd, J = 10.8, 6.2),1.31 (3H, s),4.30 (2H, overlap),7.13 (1H, br s),6.15 (1H, s),7.33 (1H, s),0.82 (3H, s),5.82 (1H, s),1.15 (3H, s),2.10 (3H, s),1.97 (3H, s),2.66–2.70 (4H, m,OCOCH_2_CH_2_COOH); ^13^C NMR (75 MHz, CDCl_3_) δ: 35.2, 73.3, 39.0, 28.0, 25.1, 69.3, 41.2, 48.3, 45.5, 207.7, 78.3, 45.5, 72.1, 58.4, 33.4, 35.2, 14.4, 64.8, 122.8, 142.3, 111.6, 140.7, 21.5, 95.1, 19.5, 171.6, 171.3, 174.3 (OCOCH_2_CH_2_COOH), 170.7 (OCOCH_2_CH_2_COOH), 28.8 (OCOCH_2_CH_2_COOH), 28.2 (OCOCH_2_CH_2_COOH); IR (KBr, ν (cm^−1^)): 3466 (vs, OH), 1730 (vs, OC = O).

### Synthesis of TSN-S Affinity Matrices

TSN-S affinity matrices were designed and prepared based on previous report (Figure S1) [17]. TSN-S (72.79 mg) and N-Hydroxysuccinimide (NHS, 24.84 mg, Shanghai Chemical Reagent Co., LTD, Shanghai, China) were dissolved in 1.2 mL dimethylformamide (DMF, Shanghai Chemical Reagent Co., LTD, Shanghai, China), and a solution of dicyclohexylcarbodiimide (44.46 mg, Shanghai Chemical Reagent Co., LTD, Shanghai, China) in 4.8 mL DMF was then added. The mixture was stirred overnight at room temperature, then centrifuged at 10000 rpm for 15 min. EAH Sepharose 4B (GE Healthcare Biosciences AB, Uppsala, Sweden) 6 mL was washed on a sintered glass filter with distilled water adjusted to pH 4.5 with HCl, followed by 0.5 M NaCl and PBS (pH 7.4) in sedimented matrices. The matrices were kept in 6 mL PBS (pH 7.4). The reaction mixture was added dropwise to the matrices, stirred for 5 h at room temperature, then filtered. The resulting matrices was washed with DMF and water, and kept in an aqueous solution of 20% ethanol until used in binding experiments. The coupling rate of TSN-S was 39.8% by determination of hydrolyzed TSN using HPLC-ELSD.

### Cell Culture

Human promyelocytic leukemia cell line (HL-60) was obtained from Type Culture Collection of Chinese Academy of Sciences (Shanghai, China). HL-60 cells were cultured in RPMI 1640 medium (Gibco, Life Technologies Corporation, USA) supplemented with 10% (v/v) heat inactivated fetal bovine serum (Gibco, Life Technologies Corporation, USA), 100 units/mL streptomycin and 100 µg/mL penicillin in a humidified 5% CO_2_ atmosphere at 37°C.

### Capture and Identification of Specific Binding Proteins

Identification of target proteins using serial affinity chromatography was performed as described previously [18]. Briefly, HL-60 cells (6×10^7^) were washed twice with ice-cold PBS and extracted in 1 mL lysis buffer A composed of 50 mM Tris-HCl, pH 7.4, 150 mM NaCl, 0.5 mM EDTA, 0.5 mM MgCl_2_, 1% Nonidet P-40 (Sunshine Bio), 0.5 mM PMSF (Biosharp), 5 µg/mL Aprotinin (Bio Basic), 5 µg/mL Leupetin (Amresco), 5 µg/ml Pepstain (Bio Basic), shaken in ice-bath for 1.5 h. After centrifugation at 16000 rpm (20000 × g), 4°C for 15 min, the supernatants were used immediately for subsequent binding experiments.

TSN-S affinity matrices (100 µL) was washed four times with 1.0 mL of lysis buffer B containing 50 mM Tris-HCl, pH 7.4, 150 mM NaCl, 0.5 mM EDTA, 0.5 mM MgCl_2_, 1% Nonidet P-40, then shaken gently with lysate from HL-60 cells in ice-bath for 40 min and precipitated by microcentrifuge at 4°C for 1 min. The resulting supernatant was mixed with another 100 µL of TSN-S affinity matrices in ice-bath, again for 40 min. The resulting matrices was washed five times with 1.0 mL of lysate buffer B, resuspended in 30 µL of SDS sample buffer containing 4% (w/v) SDS, 20% (v/v) glycerol, 0.01% (w/v) bromophenol blue, 10% (v/v) 2-mercaptoethanol, and 0.125 M Tris–HCl (pH 6.8), heated in boiling water for 5 min, and then centrifuged for 1 min. The supernatant was subjected to SDS-PAGE. The resulting bands were stained with silver staining and then comparatively analyzed to identify specific binding proteins. For reproducibility, the above procedures were carried out for multiple times.

Protein band on the SDS-PAGE gel was excised and directly digested with trypsin. Tryptic peptides were separated by nano-flow microcapillary reversed-phase HPLC and eluted directly into the LTQ- Ion trap mass spectrometer (Finnigan, Thermo Fisher Scientific Inc., USA) using a spray voltage of 3.0 KV at a capillary temperature of 170°C in positive ion mode. A full scan from m/z 400–1800 was performed in the ion trap at a resolution of 60,000. The top ten most intense ions were selected for MS/MS in the LTQ, with a normalized collision energy of 35%.

The raw data were filtered by TurboSEQUEST algorithm. Protein identification was acquired with Turbo SEQUEST (Thermo Electron) algorithm in the BioWorks 3.1 Software against the International Protein Index (IPI) human protein database (Version 3.36). Turbo SEQUEST evaluated proteins by finding candidate peptides whose masses match the m/z values in spectra within a mass error tolerance. Turbo SEQUEST used a cross-correlation scoring. Identified peptides were filtered by the Xcorr in accordance with the charge state (Charge +1,Xcorr≥1.9; Charge +2,Xcorr≥2.2; Charge +3,Xcorr≥3.75), DelCN≥0.1. The reverse database was used for false positive rate estimation to be <0.05.

### Molecular Modeling and Docking

Modeling study was performed using Accelrys Discovery Studio 2.5 (DS 2.5). The coordinates for dCK containing 2′-deoxycytidine (dC) and ADP as cocrystalized ligands were obtained from the Protein Data Bank (PDB code: 1P60). The structure of TSN was generated using ChemDraw. Docking of TSN was accomplished using CDocker [19], launched from within DS 2.5. Deviations from the default settings included generating 100 starting conformations of the ligand to adequately sample conformational space and 30 poses were selected for simulated annealing. The top scoring pose (CDOCKER_Energy) was visually inspected.

### Plasmid Construction and Site-directed Mutagenesis

Wild-type dCK and seven mutants (S35E, S35A, S35Q, S74E, S74Q, R128E and R128A ) were constructed using overlap extension PCR method and wild-type human dCK cDNA as the template [20]. The PCR products were subcloned into the expression plasmid pET28 (a+) vector providing a His-tag at the N-terminus via the 5′-Nde I/3′ECoR I sites. Then, the plasmid was transformed into *E. coli* BL21 (DE3) strain.

### Protein Expression and Purification

*E. coli* BL21 (DE3) cells harboring the pET28 (a+) expression plasmids were grown in Luria-Bertani (LB) medium at 37°C until the A_600_ reached 0.4–0.6, then induced with 0.1 mM isopropyl-β-D-thiogalactopyranoside (IPTG) about 12 h at 15°C and harvested by centrifugation [21]. After lysis by high-pressure, the supernatant was loaded onto a Ni-NTA column (GE Healthcare Biosciences AB, Uppsala, Sweden), and washed with buffer containing 0.05 M PBS (pH 7.0) and 100 mM NaCl, The enzyme was eluted by the same washing buffer containing 10 mM, 50 mM, 100 mM, 200 mM and 500 mM imidazole respectively, while NaCl was not included in washing buffer with 100 mM and 200 mM imidazole. After concentration by centrifugation, the purity of the proteins was checked by 12% SDS-PAGE. Eluted protein was used in kinase reaction.

### dCK Activity Assay

The activity of dCK was measured with Kinase-Glo® Luminescent Kinase Assay (Promega Corporation, Madison, USA) as previously described [22]. This assay determined kinase activity by quantifying the amount of ATP remaining in solution following a kinase reaction assay platform. Briefly, TSN at various concentrations were incubated with 100 nM dCK, 5 µM dC and 1 µM ATP in a 50 µL volume with 50 mM Tris (pH 7.5), 5 mM MgCl_2_, 0.5 mM DTT for 30 min at room temperature. An equal volume of Kinase-Glo® reagent was added and incubated for 10 min at room temperature. The luminescence was recorded on a LUMIstar OPTIMA reader.

### Apoptosis Assay

HL-60 cells were treated with 70 nM TSN or 0.1–10 µM LP-503392 (inhibitor of dCK, Berry & Associates, Inc., USA) alone or exposed to their binary combinations for 36 h. Then, they were harvested, washed and resuspended with PBS. Apoptotic cells were determined with an FITC Annexin V Apoptosis Detection Kit (BD Biosciences, USA) according to the manufacturer’s protocol. Annexin V^+^/PI^−^ cells were considered as apoptotic cells.

### Statistical Analysis

All experiments were repeated at least three times and the data were expressed as means ± standard deviations. Student *t* test value of *P*<0.05 was considered statistically significant.

## Results

### Synthesis of TSN Probe

Affinity matrices bearing bioactive compounds have been a useful tool in the identification of target proteins. A modified affinity chromatography, termed serial affinity chromatography, has been used to capture various target proteins. For example, the specific binding proteins of ginsenoside Rg1, FK506, benzenesulfonamide and methotrexate were successfully captured and identified by this method [18], [23]. Although TSN markedly suppressed cell growth, induced cell apoptosis and cell cycle arrest in cancer cells [10]–[13], its molecular target and mechanism remain largely unclear. In the present study, we constructed a TSN probe in order to capture the binding proteins. A TSN derivative bearing a linker moiety at C28 hydroxyl group (TSN-succinate) was first synthesized and then coupled to a commercial resin EAH Sepharose 4B (Figure S1). The structure of TSN-succinate was confirmed by comparison of the spectral data of MS, NMR and IR with those in the literature [17]. Cell viability assay showed that modifications at C28 hydroxyl group had a limited impact on the activity of TSN (data not shown). The coupling rate of TSN-S was determined by HPLC-ELSD to be 39.8%. Our data indicated that the desired TSN probe was obtained and was used for subsequent affinity chromatography.

### Capture and Identification of a Target Protein Using Serial Affinity Chromatography

**Figure 1:**
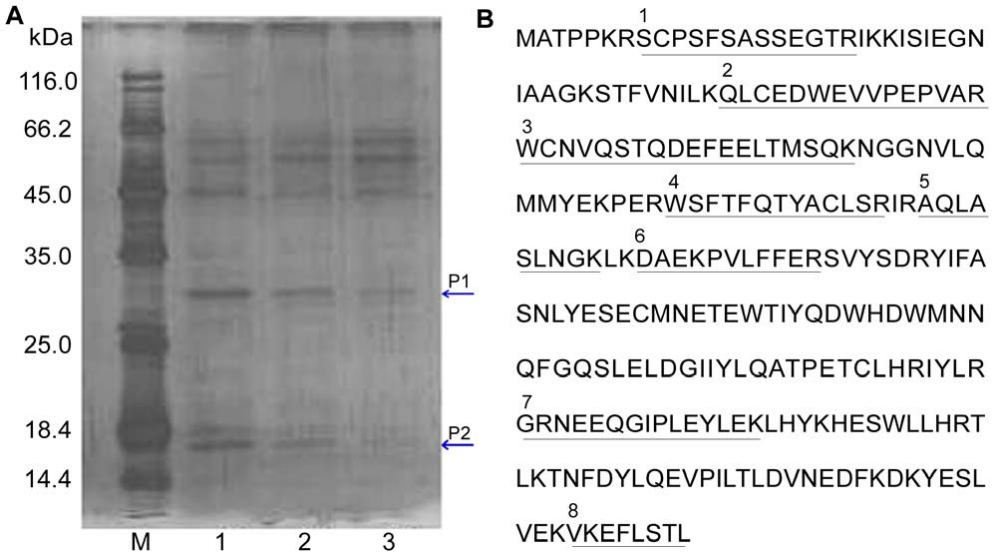
Capture and identification of dCK as a TSN-binding protein. (A) 13% SDS-PAGE for serial affinity chromatography by silver staining. Lane 1, Resin-bound proteins from the first time; Lane 2, Resin-bound proteins from the second time; Lane 3, Resin-bound proteins from the third time. Specific binding proteins are indicated as P1 and P2. (B) Amino acid sequence of human dCK. The underlined amino acid residues in dCK sequence indicate the peptides detected by tryptic digestion and MS analyses.

In the present study, HL-60 cells, the most sensitive cell line to TSN [10], were employed to investigate the mechanism of its growth inhibition. Using the TSN affinity probe, we captured specific binding proteins for TSN from cell lysates by serial affinity chromatography as shown in Figure 1A. To ascertain the specificity for the binding proteins, the same protocol was applied three times using the same affinity matrices and lysates, followed by analyzing the binding proteins captured on the matrix. The amounts of proteins P1 and P2, as indicated in the SDS-PAGE, obviously decreased following the serial affinity chromatography, whereas the amounts of other proteins remained unchanged, suggesting that proteins P1 and P2 might be specific TSN-binding proteins. MALDI-TOF-MS analysis of the band P1 showed 8 tryptic peptide sequences, all of which matched the amino acid sequence of dCK (Figure 1B, Table S1). On the other hand, the band P2 consisted of a mixture of proteins, in which only one or two tryptic peptide sequences were identified for each protein. Therefore, we concluded that dCK is a specific binding protein for TSN and focused our efforts on dCK for subsequent investigation.

### TSN Directly Activates dCK

To explore the interaction between TSN and dCK, recombinant dCK was overexpressed and purified. The dCK activity was measured using Kinase-Glo® Luminescent Kinase Assay after incubation of recombinant dCK in the presence of TSN. Different concentrations ranging from 6.25 to 250 µM TSN were tested to examine the effects to dCK activity. As depicted in Table 1, after stimulation by TSN, dCK activity was increased in the presence of 12.5–100 µM TSN compared to that of control (P<0.05), and the activation was in a dose-dependent manner. Previous studies have showed that dCK can be activated by a wide array of nucleoside analogues and genotoxic agents [24]–[30]. Regarding the mechanism of activation, a post-translational modification was suggested without change of dCK expression level. In addition, activation of dCK by several agents selectively augments the intracellular dATP pool that contributed to the formation of apoptosome and further induction of cell apoptosis [24], [31].

**Table 1:** Effects of TSN on the activity of dCK mutants.^a^

TSN (µM)	0	12.5	25	50	100
WT	100.0±2.1	106.8±1.1^b^	108.9±2.0^b^	113.3±2.3^b^	116.1±2.3^b^
S35E	100.0±1.9	97.6±1.1	98.3±2.1	99.4±2.0	97.5±2.8
S35A	100.0±2.8	98.8±3.1	96.6±5.4	99.4±3.8	98.7±3.8
S35Q	100.0±4.2	99.7±3.1	99.1±6.1	97.9±5.5	97.5±7.9
S74E	100.0±12.4	125.5±9.5^b^	134.0±11.4^b^	146.0±17.5^b^	147.7±17.7^b^
S74Q	100.0±2.0	106.1±1.9^b^	108.8±2.7^b^	112.4±2.6^b^	113.2±1.8^c^
R128E	100.0±3.2	102.3±3.3	97.9±5.0	99.5±2.4	99.0±3.0
R128A	100.0±2.6	98.6±2.2	101.7±2.7	99.7±2.4	99.1±2.5

a dCK mutants (100 nM) was incubated with indicated concentration of TSN. The activity of the enzyme was evaluated by luminescence recorded on a LUMIstar OPTIMA reader. Means and standard deviations were calculated from three independent determinations.

b *P*<0.05 vs. control (100%).

c *P*<0.01 vs. control (100%).

### dCK Inhibitor Reverses TSN-induced Apoptosis

**Figure 2:**
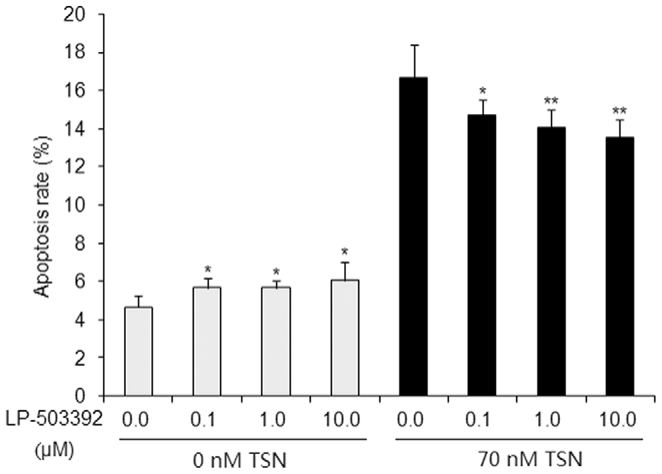
Effects of dCK inhibitor on TSN-induced HL-60 cell apoptosis. HL-60 cells were treated with indicated concentrations of LP-503392 or binary combinations of 70 nM TSN and indicated concentrations of LP-503392 for 36 h. Apoptotic effects were evaluated by Flow Cytometry analyses. Values are means ± SD.^ *^ *P*<0.05 was compared with DMSO solvent control (left panel), ^*^ *P*<0.05 and ^**^ *P*<0.01 were compared with untreated by dCK inhibitor (right panel).

To further confirm whether dCK was directly activated by TSN, a commercially available dCK inhibitor LP-503392 [32] was employed to investigate the apoptosis rate induced by TSN. By measuring the apoptotic cell rates, TSN treatment markedly induced the apoptosis of HL-60 cells [13]. While LP-503392 treatment alone showed small degree of increase on apoptotic rate (Figure 2A), addition of LP-503392 significantly reversed the apoptosis induced by TSN in a dose-dependent manner with the highest apoptosis inhibition rate up to 11.7% when HL-60 cells were exposed to the combinations of 70 nM TSN and different concentrations of LP-503392 for 36 h (Figure 2B). Although the pro-apoptotic effect of LP-503392 was unexpected, this could be due to the unknown effects by hitting other targets or pathways by this newly identified compound and the relatively low dCK activity in the cells without TSN treatments. Nevertheless, our results suggested that dCK activation at least plays certain roles in the apoptosis of HL-60 cells induced by TSN.

### Effects of TSN on the Activity of dCK Mutants

**Figure 3:**
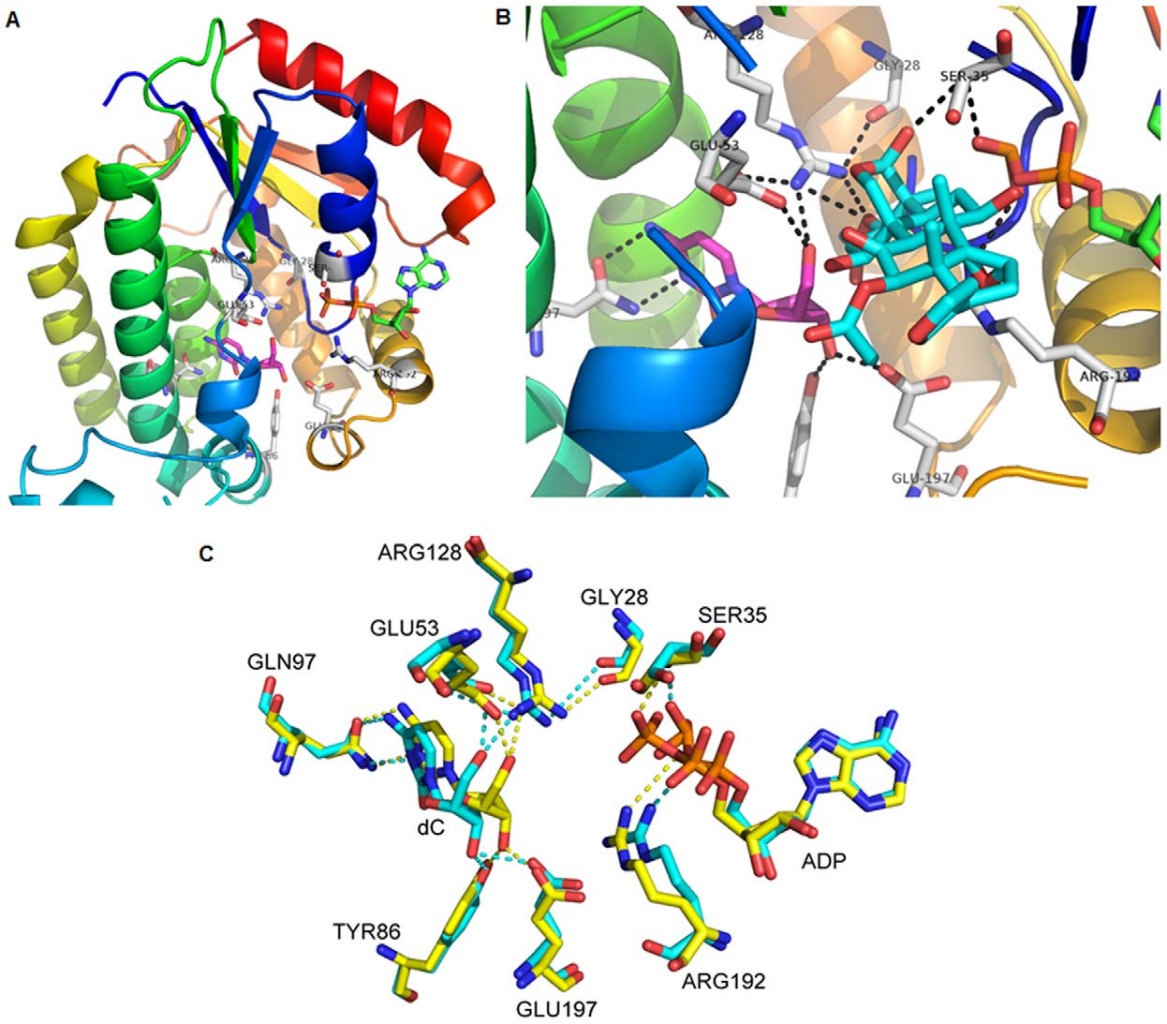
Interaction sites between TSN and dCK. (A) Structure of human dCK complexed with 2′-deoxycytidine (magenta) and ADP (green) (PDB: 1P60). The amino acids in the active site are labeled, in which Glu53 functions as a base. (B) Model of quaternary complex between dCK, 2′-deoxycytidine (magenta), ADP (green) and TSN (cyan). The amino acids and hydrogen bonds are labeled. (C) Superposition of the active-site residues of the tertiary complex (yellow) and quaternary complex (cyan). Hydrogen bonds in yellow and cyan correspond to respective structure of dCK complex. The figure was generated using PyMol.

Phosphorylation and conformational change of dCK have been suggested to lead dCK activation [24], [33]–[35]. On one hand, phosphorylation and dephosphorylation of dCK were important in the regulation of dCK activity [22]. The Ser74 residue in dCK was identified to be the only phosphorylation site that influences intracellular activity [33], [34]. To clarify whether the enhancement of dCK activity by TSN is the result of auto-phosphorylation of the enzyme, site-directed mutagenesis of Ser74 was carried out to generate three mutants of S74E, S74A and S74Q. On the other hand, conformational change of dCK could also increase its activity [24], [35]. To explore the possibility of conformational change of dCK by TSN, we modeled the quaternary complex structure of dCK-dC-ADP-TSN using the crystal structure of human dCK complexed with 2′-Deoxycytidine and ADP (PDB: 1P60) (Figure 3A). TSN positions towards the active site of dCK and is bound primarily through hydrophobic interactions consisting of residues Ile30, Ala31, Ala32, Thr36, Val55, Ala56 and Phe137. Two hydrogen bonds are formed between the TSN and the NH1 and NH2 of Arg128 (2.3 Å and 2.5 Å via C3 acetoxy group) and a single hydrogen bond is formed between the TSN and the amide hydrogen atom of Ser35 (2.4 Å via C28 hydroxyl group) as shown in Figure 3B. In dCK molecule, Glu53 is the general base to accept the proton from the 5′-hydroxyl of dC, and Arg128 interacts with the 5′-hydroxyl group of dC (2.9 Å) and with Glu53 (3.0 Å) through the NH2 group and with the carbonyl group of Gly28 (2.8 Å) via its NH1 group. Ser35 plays important role in binding and positioning the α- and β-phosphoryl groups of the phosphoryl donor via interactions between amide backbone hydrogen and phosphate oxygen atom. Given their interactions with TSN, Arg128 and Ser35 were replaced with Glu, Ala and Gln respectively to investigate the effects of TSN on dCK activity.

**Figure 4:**
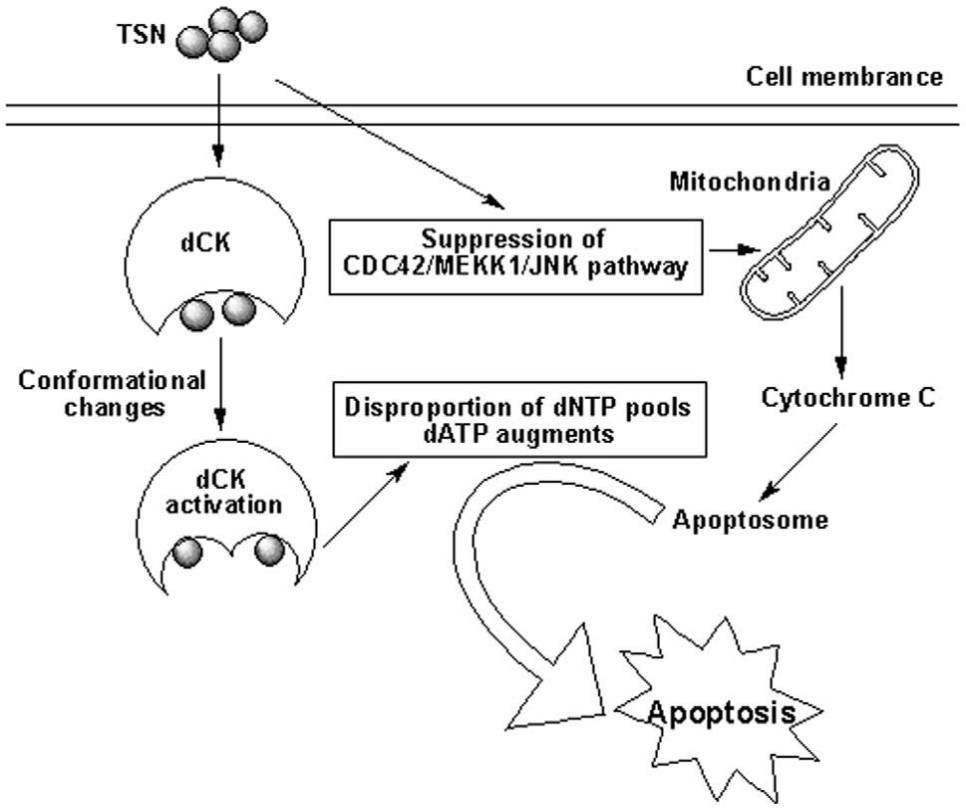
Proposed mechanism on the apoptotic effects of TSN.

After the phosphorylation site and interaction sites were replaced by site-directed mutagenesis, the mutant proteins were expressed and purified (Figure S2). To explore the effects of TSN on the activity of dCK mutants, we incubated dCK mutants with different concentration of TSN respectively. The activity of dCK mutants was measured and depicted in Table 1. After stimulation by TSN, dCK mutants S74E and S74Q could be significantly activated, whereas the activity of S35 mutants and R128 mutants was not affected. These results suggested that auto-phosphorylation at Ser74 residue could not explain dCK activation by TSN, because Ser74 mutation did not affect the activation effects by TSN. However, mutations at Ser35 and Arg128 completely diminished the activation effects by TSN. In the presence of TSN, a hydrogen-bond acceptor at the C3 acetoxy position could compete with Glu53 for the Arg128 interaction. Additionally, as shown in Figure 4C, the steric repulsion of TSN in the active site causes the dramatic drift of Glu53, Arg128 and dC, leading to the increased hydrogen-bond distance of Arg128 with Glu53 (from 3.0 Å to 3.8 Å). Hence, a weaker Glu53–Arg128 interaction would facilitate the proton-accepting ability of the carboxylic acid group from the O5′-hydroxyl. As a result, the nucleoside 5′-hydroxyl group would become more nucleophilic to attack to ATP γ-phosphate, thereby the enzymatic activity for dC phosphorylation would be increased. On the other hand, TSN simultaneously interacts with the 5′-hydroxyl group of dC (2.1 Å) via C3 acetoxy oxygen atom and with α- and β-phosphate of ADP via its O-10 and O-14 (2.4, 1.9, 2.3 and 2.1 Å). Collectively, TSN would act as a clamp to bring the 5′-hydroxyl group of dC close to the γ-phosphate of ATP. Furthermore, Ser35 could play an important role in anchoring the position of activator and phosphoryl donor by directly interacting with TSN (3.2 Å) and ATP (2.8 Å).

## Discussion

Naturally occurring small molecules are important tools for cell biology and are lead molecules for drug discovery. Identification of the molecular targets of natural products has had profound influence on studies of complex cellular machinery [14]. Although TSN markedly suppressed cell growth, induced cell apoptosis and cell cycle arrest in cancer cells 10–13, its mechanism was largely unclear prior to the present study. In this study, we have constructed TSN probe to capture specific binding proteins, and a specific binding protein was identified to be dCK.

dCK (EC 2.7.1.74) is a rate-limiting enzyme in the salvage pathway of DNA synthesis. The enzyme phosphorylates deoxyadenosine, deoxyguanosine and deoxycytidine, with UTP or ATP as phosphate donor [36], [37]. In addition, it is responsible for the phosphorylation of several clinically important antiviral and antineoplastic nucleoside analogues [38]–[40]. The level of dCK activity is one of the major factors determining the sensitivity of different leukemia and solid tumors to deoxynucleoside analogue toxicity [9], [38]. Furthermore, dCK is required for T and B lymphocyte development in the thymus and bone marrow. Lymphocyte numbers in the dCK KO mice are 5- to 13- fold below normal values. A 90-fold decrease in the total number of thymocytes is observed in the dCK KO mice relative to wild-type littermates [41]. Previous studies have demonstrated that dCK could be activated by various nucleoside analogues and genotoxic agents such as CdA [6], [24]. Inhibitors of topoisomerase II and DNA polymerase [27], [28], UV- and γ-irradiation [29], [30], as well as other compounds including protein phosphatase inhibitors and etoposide [27]. The activation of dCK could alter the composition of dNTP pools, selectively augmenting intracellular dATP level that contributes to the formation of apoptosome and further induction of cell apoptosis [1], [24]. In the present study, although the dCK activation by TSN was modest, this direct activation may contribute, in part, to the cell apoptosis through either changing the dATP level or participating in other signaling pathways. It is generally known that cell apoptosis is a complex process, which involves multiple steps and signaling pathways. In our previous study, TSN could induce HL-60 cell apoptosis by suppressing JNK signaling pathway [13]. The direct activation of dCK by TSN and the reverse of apoptotic rate by a dCK inhibitor LP-503392 in the presence of TSN in this study suggest that dCK activation may partially contribute to the cell apoptosis induced by TSN in addition to the suppression of JNK signaling. Moreover, even though dCK is thought to participate in the salvage pathway of DNA synthesis, the present results further imply that dCK could play more complicated roles through interacting with other proteins or signaling pathways in the apoptotic process of tumor cells.

It is known that Ser74 residue is the only phosphorylation site that influences dCK activity [33], [34]. To identify whether the enhanced effect of TSN on dCK activity is the result of an auto-phosphorylation of the enzyme, site-directed mutagenesis of Ser74 was performed. Our findings showed that dCK mutants (S74E and S74Q) could still be activated significantly by TSN. The results ruled out the possibility that dCK activation by TSN was caused by the auto-phosphorylation of Ser74. Based on previous studies that activation of dCK involves a conformational change [24], [35], we simulated the interaction between TSN and dCK, and two potential interaction sites (Ser35 and Arg128) were identified. After mutagenesis, Ser35 and Arg128 mutants diminished the activation from TSN, indicating that the increase of dCK activity is caused by its conformational changes.

In the present study, the effective concentrations of TSN towards recombinant dCK and HL-60 cells were significantly different. This difference indicates that dCK activation only plays certain and limited roles in the HL-60 cell apoptosis, and TSN, a known cytotoxic compound, acts on multiple targets and pathways to induce the complex apoptotic process. In addition, the relatively weak activation effects by TSN on dCK activity may amplify to interfere the biological process and to interact with other proteins and signaling systems for the final apoptotic effects in HL-60 cells. Therefore, combination of the present study and previous findings [13] leads us to propose a new route, in which the apoptotic effects of TSN are partially mediated by the interaction between TSN and dCK to result in a conformational change of dCK, leading to the elevation of dCK activity. Subsequently, activation of dCK by TSN selectively augments the intracellular dATP pool that contributes to the formation of apoptosome and further induction of cells apoptosis (Figure 4).

Since TSN possesses a variety of biological activities, these different activities should be a result of targeting multiple proteins or signaling pathways. From the apoptosis point of view, the interference on the complex network might be responsible for the final outcomes. The present findings indicate that TSN can bind dCK and activate the enzyme through conformational changes, which at least explains, in part, a new apoptotic route for TSN. The exact network or signaling systems by which TSN interacts with different proteins requires further analysis.

## Supporting Information

Figure S1 **The synthetic route of TSN-S affinity matrices.**
(TIF)

Figure S2 **13% SDS-PAGE of purified dCK mutants.** Bands 1–8 represent dCK mutants of S35E, S35A, S35Q, S74E, S74Q, R128E, R128A and WT dCK respectively. M indicates molecular weight markers.
(TIF)

Table S1 **Theoretical and detected molecular masses of amino acid sequences of human dCK.**
(DOC)
