# Supplementary figures and images for: The Apoptotic Effects of Toosendanin Are Partially Mediated by Activation of Deoxycytidine Kinase in HL-60 Cells
Source: PLoS One. 2012 Dec 27;7(12):e52536. doi: 10.1371/journal.pone.0052536 (PMC3531419; doi:10.1371/journal.pone.0052536)

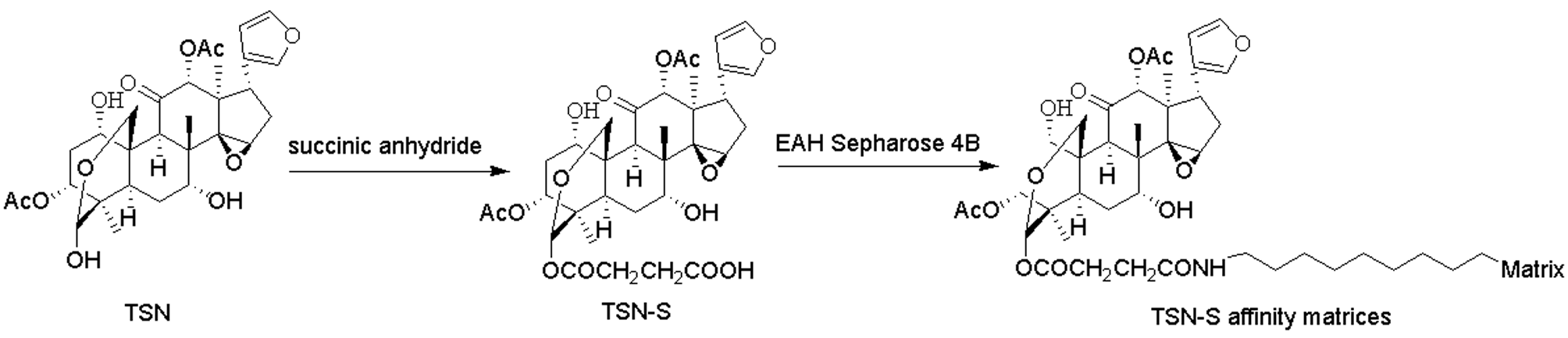

Supplement: Figure S1 — The synthetic route of TSN-S affinity matrices. (TIF) [file pone.0052536.s001.tif]

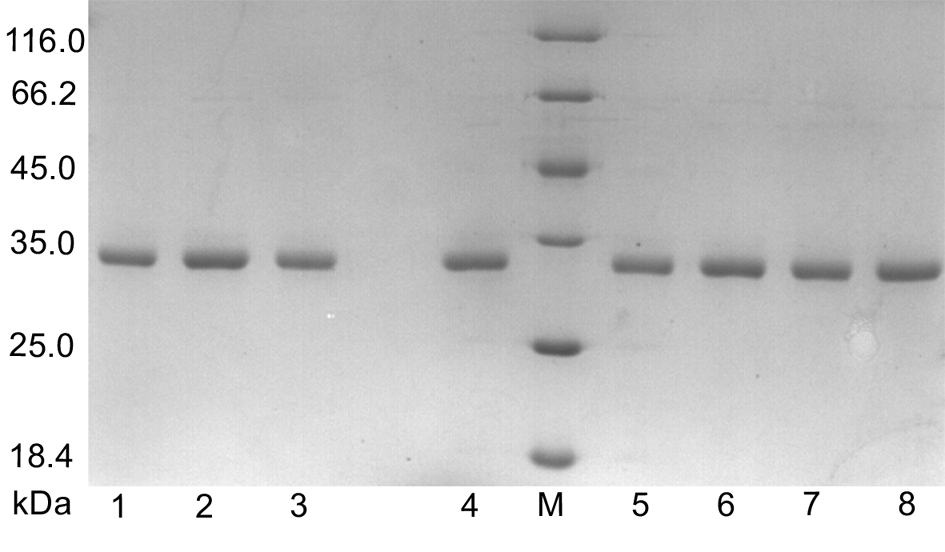

Supplement: Figure S2 — 13% SDS-PAGE of purified dCK mutants. Bands 1–8 represent dCK mutants of S35E, S35A, S35Q, S74E, S74Q, R128E, R128A and WT dCK respectively. M indicates molecular weight markers. (TIF) [file pone.0052536.s002.tif]
