# Supplementary material for: The Apoptotic Effects of Toosendanin Are Partially Mediated by Activation of Deoxycytidine Kinase in HL-60 Cells
Source: PLoS One. 2012 Dec 27;7(12):e52536. doi: 10.1371/journal.pone.0052536 (PMC3531419; doi:10.1371/journal.pone.0052536)
Supplement: Table S1 — Theoretical and detected molecular masses of amino acid sequences of human dCK. (DOC) [file pone.0052536.s003.doc]

**Table S1.** Theoretical and detected molecular masses of amino acid sequences of human dCK.

| No. | Amino acid sequence | Theoretical value (Da) | Detected value (Da) |
| --- | --- | --- | --- |
| 1 | scpsfsassegtr | 1373.4432 | 1373.4016 |
| 2 | qlcedwevvpepvar | 1828.0562 | 1828.0090 |
| 3 | wcnvqstqdefeeltmsqk | 2361.5727 | 2361.5212 |
| 4 | wsftfqtyaclsr | 1667.8857 | 1667.8389 |
| 5 | aqlaslngk | 902.0388 | 902.0307 |
| 6 | daekpvlffer | 1351.5453 | 1351.5320 |
| 7 | grneeqgipleylek | 1775.9568 | 1775.9407 |
| 8 | vkeflstl | 937.1243 | 937.1149 |
